# Real-World Evaluation of the Efficacy and Tolerability of a Fixed-Dose Combination of Amlodipine and Indapamide in Patients Over 55 Years

**DOI:** 10.7759/cureus.92756

**Published:** 2025-09-19

**Authors:** Quazi Tarikul Islam, KMHS Sirajul Haque, Kaniz Moula, Khan Abul Kalam Azad, H A M Nazmul Ahasan, Md. Mujibur Rahman, Md. Ismail Patwary, Zakir Hossain, Abul Kalam Azad, Titu Miah

**Affiliations:** 1 Department of Medicine, Popular Medical College, Dhaka, BGD; 2 Department of Cardiology, Anwer Khan Modern Medical College, Dhaka, BGD; 3 Department of Medicine, United Hospital Limited, Dhaka, BGD; 4 Department of Medicine, Bangabandhu Sheikh Mujib Medical University, Dhaka, BGD; 5 Department of Medicine, Sylhet Women's Medical College, Sylhet, BGD; 6 Department of Medicine, TMSS Medical College, Bogura, BGD; 7 Department of Medicine, Dhaka Medical College Hospital, Dhaka, BGD

**Keywords:** amlodipine, blood pressure control, elderly hypertension, indapamide, isolated systolic hypertension

## Abstract

Background: Isolated systolic hypertension (ISH) is a major cardiovascular risk factor in older adults, yet blood pressure (BP) control remains suboptimal. This study assessed the real-world effectiveness and tolerability of a FDC of indapamide and amlodipine in Bangladeshi patients aged 55 years and older.

Methods: The COMBINE (COMBination of Indapamide and amlodipiNE) study was an open-label, multicenter, prospective observational study conducted across 10 tertiary centers in Bangladesh from June to October 2022. A total of 185 patients with ISH, defined as systolic BP (SBP) between 140 and 179 mmHg (millimeters of mercury), received indapamide 1.5 mg and amlodipine 5 mg once daily. Primary endpoints included the mean SBP change and the proportion achieving SBP targets (<140 mmHg, <130 mmHg). BP, adverse events, and patient-reported well-being were assessed using the visual analogue scale (VAS).

Results: The mean SBP decreased by 20.9, 24.7, and 29.3 mmHg (p < 0.001); mean diastolic BP (DBP) declined by 8.1, 10.4, and 12.9 mmHg (p < 0.001) at two, four, and 12 weeks, respectively. At 12 weeks, most patients (164, 88.6%) achieved SBP <140 mmHg, and over half (115, 62.2%) of patients reached SBP <130 mmHg. Pulse pressure also improved significantly. The treatment was well tolerated, with mild adverse events including leg edema in three (1.6%) and hypokalemia in four (2.2%). VAS scores improved significantly, reflecting enhanced patient-perceived health status.

Conclusions: In his real-world study, therapy was associated with substantial BP reductions, target SBP achievement, good tolerability, and improved patient-reported well-being in older adults with ISH.

## Introduction

Hypertension is a major modifiable risk factor for cardiovascular disease (CVD), chronic kidney disease (CKD), and all-cause mortality, with a dose-dependent (log-linear) relationship that persists independent of other risk factors, particularly notable in older adults [1,2]. In this population, hypertension is not only highly prevalent but also linked to a greater risk of dementia, physical disability, and an increased likelihood of falls and fractures [3].

Among elderly individuals, isolated systolic hypertension (ISH) represents the most common subtype of hypertension [4]. Definitions vary across guidelines: the American guidelines classify ISH as systolic blood pressure (SBP) ≥130 mmHg (millimeters of mercury) with diastolic blood pressure (DBP) <80 mmHg, while European and most other guidelines define ISH as SBP ≥140 mmHg and DBP <90 mmHg [5,6]. Data from the National Health and Nutrition Examination Survey (1999-2010) highlight its burden, reporting that 30% of adults aged 60 years and older have untreated ISH, compared with 6% of those aged 40-50 years and only 1.8% of individuals aged 18-39 years [7]. The pathophysiology of ISH is primarily driven by arterial stiffening, increased vascular resistance, and endothelial dysfunction, which collectively heighten the risk of stroke, heart failure, and renal impairment [8,9].

Despite the clinical importance of ISH, blood pressure control in elderly patients remains suboptimal [4]. Evidence consistently shows that lowering SBP below recommended thresholds significantly reduces cardiovascular events and all-cause mortality [10]. Antihypertensive therapy is therefore central to improving outcomes in this population [10]. However, the choice of therapy is important, as drug classes differ in their effects on arterial stiffness and pulse wave augmentation. β-blockers, although no longer recommended as first-line therapy, primarily lower SBP by acting on the first component of the pulse wave [11]. By contrast, calcium channel blockers (CCBs), diuretics, and angiotensin II inhibitors exert more favorable effects on vascular compliance [12].

Hypertension is highly prevalent in Bangladesh; national survey data estimate that approximately 28% of adults are hypertensive, with about one in eight of these being undiagnosed [13]. While prevalence increases with age, specific data on ISH in older populations in Bangladesh remain limited. Despite the high burden, treatment and control rates remain suboptimal in Bangladesh; evidence suggests that only about one-third of individuals with hypertension achieve target blood pressure levels (BP control rate ~33%) [14]. A prescribing pattern study from a tertiary hospital in India demonstrated that CCB+ARB dual therapy was the most commonly used two-drug combination, followed by ARB+diuretic regimens [15]. However, fixed-dose combinations (FDCs) comprising indapamide plus a CCB, as used in our study, remain notably underrepresented in standard prescribing practices - highlighting the novelty and clinical relevance of our investigation.

Against this background, the present study evaluated the real-world effectiveness and tolerability of a FDC of amlodipine and indapamide (NATRIXAM) in Bangladeshi patients aged 55 years and older with ISH, defined as SBP ≥140 mmHg and DBP <90 mmHg. NATRIXAM is a single-pill formulation containing indapamide sustained release 1.5 mg and amlodipine 5 mg, a dihydropyridine CCB, indicated for the management of hypertension, particularly ISH in older adults [16]. Although ISH is a pressing public health concern, evidence regarding its management in Bangladesh remains limited, especially among older populations. Most clinical trials of antihypertensive agents have been conducted in Western settings, which may limit their applicability to South Asia. Considering the unique genetic, dietary, and healthcare system factors in Bangladesh, this study was designed to generate region-specific, real-world evidence on the use of this FDC.

The primary objective was to assess the changes in SBP, focusing on mean SBP reduction from baseline to 12 weeks and the proportion of patients achieving target SBP levels of <140 mmHg and <130 mmHg over the study period. Secondary objectives included evaluating changes in DBP and pulse pressure (PP), exploring age-related variations in treatment response, monitoring safety outcomes such as adverse events and serum electrolyte changes (particularly potassium), and assessing patient-reported quality of life using the visual analogue scale (VAS). Blood pressure and other outcomes were assessed at baseline and during follow-up visits at weeks two, four, and 12.

This article has been presented at the ESH Congress 2023 in Berlin, Germany, and the abstract has been published in the Journal of Hypertension (10.1097/01.hjh.0001022220.64710.64).

## Materials and methods

Study design and settings

This COMBINE (COMBination of Indapamide and amlodipiNE) study is an open-label, multicenter, uncontrolled, prospective observational study conducted at 10 tertiary healthcare centers in Bangladesh from June to October 2022. The centers were randomly selected using stratified random sampling to ensure geographical diversity across three divisional cities, including both urban and semi-urban healthcare facilities. Given the real-world observational nature of the study, no comparator arm was included, allowing evaluation under routine clinical practice.

Study population

The inclusion and exclusion criteria are summarized in Table 1.

**Table 1:** Inclusion and exclusion criteria for study enrollment the COMBINE study SBP: systolic blood pressure; DBP: diastolic blood pressure; ISH: isolated systolic hypertension; PP: pulse pressure; NYHA: New York Heart Association; COMBINE: COMBination of Indapamide and amlodipiNE Note: Although the classical definition of ISH specifies DBP <90 mmHg, a subset of older adults with SBP ≥140 mmHg and PP ≥50 mmHg was included, even when the DBP was marginally above 90 mmHg. This approach aimed to capture clinically relevant ISH phenotypes associated with arterial stiffness and elevated cardiovascular risk, reflecting real-world presentations.

Inclusion Criteria	Exclusion Criteria
Age 55 years and older	Age less than 55 years
Confirmed diagnosis of primary arterial hypertension (previously diagnosed/treated or newly identified)	Severe hypertension: SBP ≥180/110 mmHg while on treatment or ≥200/110 mmHg if untreated
For previously treated patients: completion of a 7-day washout or supervised titration of drug omission/addition before study therapy initiation	Resistant hypertension (requiring three or more antihypertensive agents, including a diuretic, at maximum tolerated doses)
Grade 1–2 ISH: SBP 140–179 mmHg or ≥130 mmHg if uncontrolled in preceding 3 months	Symptomatic orthostatic hypotension
Pulse pressure (PP) ≥50 mmHg	Recent major cardiovascular event (myocardial infarction, unstable angina, stroke, or transient ischemic attack within the past 6 months)
Patients with SBP ≥140 mmHg and PP ≥50 mmHg were included even if DBP was marginally >90 mmHg	NYHA class III-IV congestive heart failure
Both previously treated patients with uncontrolled hypertension and treatment-naïve patients were eligible	Already taking indapamide and/or amlodipine in previous treatment
Agreed to participate in the study and provided written informed consent	Contraindications to indapamide or amlodipine based on standard clinical guidelines

Sample size calculation

The sample size was calculated to detect a minimum clinically significant reduction of 5 mmHg in systolic blood pressure (SBP) with 90% power and a two-sided significance level of 0.05. The standard formula for a one-sample mean test was applied:

$n=\frac{{(Z_{\\\alpha/2}+Z_{\beta})^{2}&times;\sigma^{2}}}{\delta^{2}}$

where Zα/2 = 1.96 for a 95% confidence level; Zβ = 1.28 for a 90% power; σ = 10 mmHg, based on variability reported in the ARBALET study evaluating indapamide/amlodipine combination in older patients with isolated systolic hypertension [17]; and δ = 5 mmHg as the minimum clinically significant difference. This calculation yielded a minimum required sample size of 42 participants. After adjusting for an anticipated 10% dropout rate, the required sample size increased to approximately 47 participants. However, to improve external validity and allow for subgroup analyses, a larger cohort was targeted, resulting in the enrollment of 213 patients, with 185 completing the study. This final sample far exceeded the minimum requirement, ensuring robust statistical power and allowing for age-stratified analyses.

Study procedures

Baseline Assessment

Patients were screened during routine outpatient visits, where investigators conducted a comprehensive evaluation to determine eligibility for study participation. A detailed demographic and clinical history was recorded, including comorbidities, previous antihypertensive therapies, and cardiovascular risk factors. Two consecutive blood pressure readings were obtained from the same arm at each visit using appropriately sized cuffs, after a five-minute seated rest, following the Korotkov method with validated digital BP monitors (OMRON HEM-7120). The average of the two readings was recorded as the patient’s BP. Standardized standard operating procedures (SOPs) and centralized investigator training were implemented across all 10 study sites to ensure consistent measurement techniques and minimize variability. Baseline laboratory investigations included fasting plasma glucose, glycated hemoglobin (HbA1c), serum creatinine, estimated glomerular filtration rate (eGFR), and serum electrolytes (sodium and potassium). Additional assessments included lipid profile (total cholesterol, low-density lipoprotein cholesterol (LDL-C), high-density lipoprotein cholesterol (HDL-C, triglycerides), hepatic enzymes (alanine aminotransferase (ALT), aspartate aminotransferase (AST), and serum uric acid. The visual analogue scale (VAS) was used to capture patient-reported well-being and perceived treatment tolerability throughout the study, providing supplementary information on perceived tolerability and symptom burden. The VAS consisted of a 10-cm horizontal line, where 0 represented “worst possible health status” and 10 represented “best possible health status.” Participants marked their overall sense of well-being at baseline and each follow-up visit, providing a subjective measure that complemented objective clinical and laboratory outcomes. All participants received detailed study information and provided written informed consent before enrollment.

Treatment approach

All enrolled patients received a FDC of indapamide (1.5 mg) and amlodipine (5 mg) (NATRIXAM) administered once daily in the morning at a consistent time, as recommended in prescribing guidelines. Morning dosing was chosen to optimize adherence and minimize nocturia due to the diuretic component; the medication could be taken with or without food.

Patients previously treated with antihypertensive drugs underwent a supervised seven-day washout to eliminate residual pharmacologic effects before initiating the study medication. The FDC was introduced as first-line therapy for treatment-naïve patients. This regimen was chosen for its documented efficacy in managing ISH in older adults by simultaneously addressing vascular resistance and arterial stiffness. All previously prescribed antihypertensive medications were discontinued during the seven-day supervised washout period before starting the FDC. Throughout the study, no additional antihypertensive agents were introduced or adjusted. Therapy adherence was enhanced through counseling, pill counts, and diary monitoring.

Follow-up and data collection

Follow-up visits were scheduled at two weeks, four weeks, and 12 weeks. The interim visits at two and four weeks primarily aimed to monitor safety, adherence, and early changes in blood pressure (BP), while the 12-week visit was designated as the primary endpoint for evaluating full therapeutic efficacy, consistent with pharmacodynamic expectations of the fixed-dose indapamide/amlodipine therapy. At each visit, BP and heart rate (HR) were measured under standardized conditions, and patient-reported well-being was assessed using the VAS. Any adverse events or unexpected clinical symptoms were systematically documented. Adherence and treatment response were monitored throughout the study. Patients were provided with digital BP monitors (OMRON HEM-7120) and instructed to record their BP and HR measurements twice daily (morning and evening) in home diaries. Investigators reviewed these data at scheduled follow-ups and adjusted management as clinically indicated. As the primary endpoint, the 12-week visit included a review of available laboratory results to assess potential metabolic or renal function changes related to therapy. Data from all visits were analyzed to determine the overall efficacy, safety, and tolerability of the intervention.

Outcome measures

Primary Outcomes 

The primary endpoints were the proportion of patients achieving systolic BP (SBP) targets of <140 mmHg and <130 mmHg from baseline to 12 weeks, as well as the mean change in SBP over the study period.

Secondary Outcomes

Secondary endpoints were defined to provide a broader assessment of cardiovascular efficacy and safety. Changes in diastolic BP (DBP) and pulse pressure (PP) were evaluated at baseline, two weeks, four weeks, and 12 weeks. An age-stratified analysis of SBP target achievement (<140 mmHg and <130 mmHg) was also performed across predefined age groups (55-59, 60-69, 70-79, and ≥80 years) as exploratory assessments to identify potential differences in treatment response among these age groups.

Safety Assessments

Safety assessments focused on adverse events typically associated with calcium channel blocker and thiazide-like diuretic therapy, including leg edema, electrolyte imbalance, and symptomatic hypotension. Laboratory tests, including serum sodium, potassium, and creatinine, were performed at baseline and at the 12-week follow-up visit to monitor for potential metabolic or renal effects. Hypokalemia was defined as serum potassium <3.5 mmol/L. Leg edema was assessed at each visit by physical examination, using standard clinical criteria for pitting edema. Findings were documented in case report forms and categorized as either absent or present.

Patient-Reported Well-Being

Patient well-being was evaluated using the VAS at baseline and at each follow-up visit, providing a subjective measure of health status to complement objective clinical and laboratory findings.

Statistical analysis

All statistical analyses were performed using descriptive and inferential methods to evaluate the effectiveness and safety of the indapamide/amlodipine combination therapy.

The primary endpoints were as follows: (a) the mean change in clinic SBP from baseline to 12 weeks and (b) the proportion of patients achieving prespecified SBP targets (<140 mmHg and <130 mmHg) at 12 weeks. Secondary endpoints included changes in DBP and PP over time, age-stratified analyses of SBP target achievement, safety outcomes (adverse events and laboratory changes), and patient-reported well-being measured by the VAS.

The full analysis set (FAS), analogous to the intention-to-treat population, included all participants who received at least one dose of study medication and had both baseline and at least one post-baseline BP measurement. The per-protocol set included participants who completed the 12-week follow-up without major protocol deviations. Since all 185 enrolled patients completed the study without attrition or significant deviations, the FAS and PP populations were the same.

Continuous variables were summarized as mean ± standard deviation (SD), and categorical variables as counts (percentages). Within-subject changes from baseline were evaluated using paired t-tests, with corresponding two-sided 95% confidence intervals (CIs) for mean differences. Analyses were conducted on the full cohort (n = 185). Exact p-values (to three decimal places, except when p < 0.001) and 95% CIs were reported for all main outcomes. Proportions were compared using chi-square tests or Fisher’s exact test where appropriate. Comparisons between groups, such as different age strata, were conducted using one-way ANOVA.

To account for repeated BP measurements, a linear mixed-effects model with random intercepts for subjects (and sites, where appropriate) was applied to address within-subject correlation and validate the robustness of paired-test findings. Results from mixed models are reported alongside paired-test results where relevant.

To address missing data, the last observation carried forward (LOCF) method was utilized for primary and secondary endpoints when follow-up data were unavailable. LOCF was selected because it is a widely used imputation technique in real-world observational studies with minimal dropout. This method was deemed appropriate given the short follow-up duration of 12 weeks and the low proportion of missing data, which helped maintain the sample size for statistical comparisons. However, we acknowledge that LOCF may slightly overestimate treatment effects. To address this, a sensitivity analysis excluding imputed cases was performed, producing consistent results that support the robustness of the findings. Statistical analysis was conducted using the Statistical Product and Service Solutions (SPSS, version 25; IBM SPSS Statistics for Windows, Armonk, NY).

## Results

Baseline characteristics

**Figure 1:**
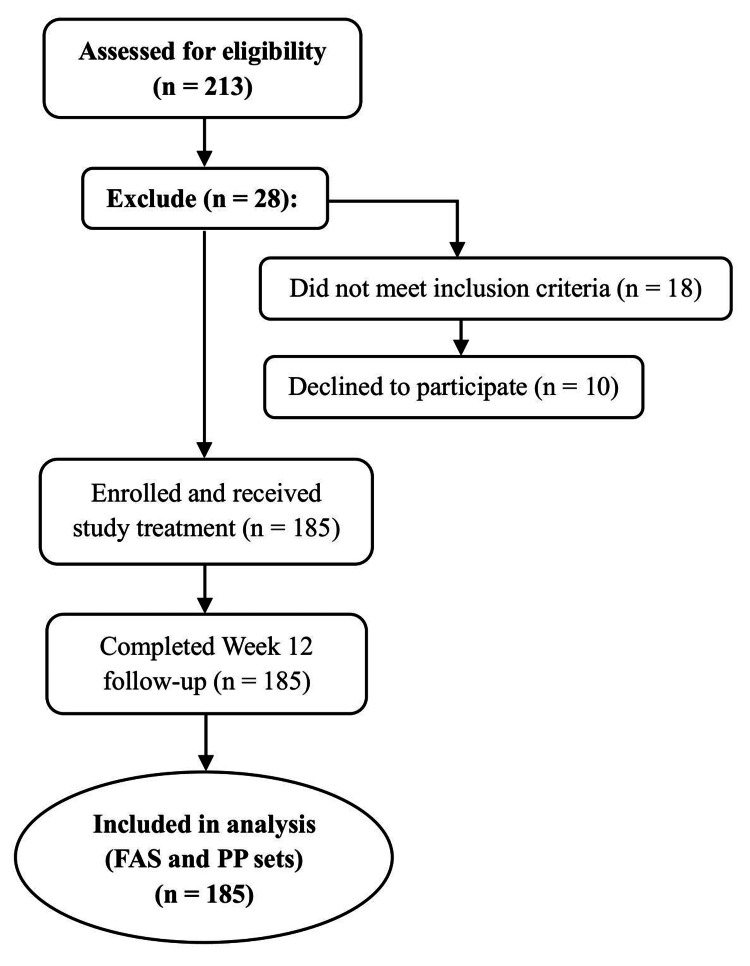
Flow diagram of patient enrollment and analysis Of the 210 individuals screened, 25 were excluded. The remaining 185 participants received treatment, completed the 12-week follow-up, and were included in the final analysis. All 185 participants were incorporated into both the full analysis set (FAS) and per-protocol analyses.

A total of 213 patients with ISH were initially enrolled in the COMBINE study across 10 distinct centers in Bangladesh. However, 185 patients completed the study, and their sociodemographic characteristics, risk factors, baseline blood pressure, and comorbidities were analyzed (Figure 1).

Among the 185 study participants, women comprised the majority, with 109 (58.9%), while men accounted for 76 (41.1%). The mean age was 62.4 ± 7.4 years, ranging from 55 to 85 years. The largest age group was 55-59 years, comprising 84 (45.4%) of patients. Cardiovascular risk factors were common: 26 (14.1%) of patients were current smokers, and 21 (11.4%) reported a family history of cardiovascular disease. The most common concomitant conditions included type 2 diabetes mellitus in 74 (40.0%) of patients, dyslipidemia in 62 (33.5%), chronic obstructive pulmonary disease (COPD)/asthma in 27 (14.6%), and coronary artery disease (CAD) in 20 (10.8%). Fewer participants had a prior history of stroke or transient ischemic attack (TIA), with six (3.2%) of patients affected; only one (0.54%) had peripheral artery disease, and two (1.1%) had undergone coronary revascularization. At baseline, blood pressure was elevated, with a mean systolic value of 156.7 ± 19.7 mmHg, a mean diastolic value of 88.5 ± 11.9 mmHg, and a mean pulse pressure of 67.9 ± 12.3 mmHg (Table 2).

**Table 2:** Baseline characteristics of patients with ISH (n = 185) SD: standard deviation; CVD: cardiovascular disease; TIA: transient ischemic attack; COPD: chronic obstructive pulmonary disease; mmHg: millimeters of mercury Values are expressed as frequency (percentage) or mean ± SD, as appropriate. Blood pressure values are presented in mmHg.

Variable	n (%) or (Mean ± SD)
Sex	
Men	76 (41.1)
Women	109 (58.9)
Age group (Years)	
55–59	84 (45.4)
60–69	63 (34.1)
70–79	30 (16.2)
80 years and older	8 (4.3)
Age (mean ± SD) years	62.4 ± 7.4
Range (min-max) years	55–85
Risk factors	
Current smoker	26 (14.1)
Family history of CVD	21 (11.4)
Concomitant diseases and conditions	
CAD	20 (10.8)
Dyslipidemia	62 (33.5)
History of coronary revascularization	2 (1.1)
History of stroke or TIA	6 (3.2)
Peripheral artery disease	1 (0.54)
Type 2 diabetes mellitus	74 (40.0)
COPD/Asthma	27 (14.6)
Blood pressure (mean ± SD) mmHg	
Systolic blood pressure	156.7 ± 19.7
Diastolic blood pressure	88.5 ± 11.9
Pulse pressure (PP)	67.9 ± 12.3

Baseline biomarkers

At baseline, the mean electrolyte balance, with normal serum potassium, was 4.1 ± 0.5 mmol/L, and the sodium concentration was 137.9 ± 3.4 mmol/L. A stable average serum creatinine level was found, at 1.0 ± 0.3 mg/dL. The mean eGFR and HbA1c levels were 71.4 ± 18.2 mL/min/1.73 m² and 6.9 ± 1.6%, respectively. Regarding lipid parameters, the mean total cholesterol was 208.2 ± 75.1 mg/dL, with LDL-C averaging 125.6 ± 66.9 mg/dL. HDL-C had a mean value of 44.8 ± 11.7 mg/dL. The mean triglyceride concentration was 199.2 ± 108.6 mg/dL (Table 3).

**Table 3:** Baseline biomarkers of study patients (n = 185) SD: standard deviation; mmol/L: millimoles per liter; mg/dL: milligrams per deciliter; HbA1c: glycated hemoglobin; eGFR: estimated glomerular filtration rate; LDL-C: low-density lipoprotein cholesterol; HDL-C: high-density lipoprotein cholesterol Values are expressed as mean ± SD.

Variable	Mean ± SD
Potassium (mmol/L)	4.1 ± 0.5
Sodium (mmol/L)	137.9 ± 3.4
Serum creatinine (mg/dL)	1.0 ± 0.3
HbA1c (%)	6.9 ± 1.6
eGFR (mL/min/1.73 m²)	71.4 ± 18.2
Total cholesterol (mg/dL)	208.2 ± 75.1
LDL-C (mg/dL)	125.6 ± 66.9
HDL-C (mg/dL)	44.8 ± 11.7
Triglycerides (mg/dL)	199.2 ± 108.6

Concomitant treatments

Among the 185 patients included in the study, a total of 102 (55.1%) had a history of prior antihypertensive medication use, while 83 (44.9%) were treatment-naive at the time of enrollment. Among 102 previously treated, angiotensin II receptor blockers (ARBs) were the most commonly prescribed monotherapy, accounting for 59 (57.8%) of patients, followed by calcium channel blockers (CCBs) in 14 (13.7%), beta-blockers (BBs) in 10 (9.8%), angiotensin-converting enzyme inhibitors (ACEi) in six (5.9%), and diuretics in five (4.9%) of patients. Additionally, only one (1.0%) had been prescribed an imidazoline receptor agonist.

A small proportion of seven (6.9%) had received dual therapy, which included combinations of ARB with diuretics (ARB+D), ARB with CCBs (ARB+CCB), or CCBs with beta-blockers (CCB+BB). However, none of these patients had been on an indapamide/amlodipine single-pill combination (SPC) before the study (Table 4).

**Table 4:** Previously prescribed concomitant antihypertensive drugs (n = 102) Values are presented as frequency (percentage). Monotherapy and dual-therapy patterns are displayed for patients who had received antihypertensive treatment before enrollment

Name of Drug	n (%)
Monotherapy (n = 95)	
Angiotensin II Receptor Blockers (ARB)	59 (57.8)
Calcium Channel Blockers (CCB)	14 (13.7)
Beta-Blockers (BB)	10 (9.8)
ACE Inhibitors (ACEi)	6 (5.9)
Diuretics (D)	5 (4.9)
Imidazoline Receptor Agonist	1 (1.0)
Dual Therapy (n = 7)	
ARB + D	7 (6.9)
ARB + CCB	
CCB + BB	

Effectiveness of indapamide/amlodipine combination therapy

Over the 12-week follow-up period, patients receiving the FDC therapy exhibited a significant and progressive reduction in both systolic and diastolic blood pressure (p < 0.001 at all time points). At two weeks, the mean SBP decreased from 156.7 ± 19.7 mmHg at baseline to 135.8 ± 18.9 mmHg, reflecting a mean change of -20.9 mmHg (95% CI: -22.1 to -19.7; p < 0.001). During the same period, mean DBP declined from 88.5 ± 11.9 mmHg to 80.4 ± 10.8 mmHg, a change of -8.1 mmHg (95% CI: -9.3 to -7.0; p < 0.001). By four weeks, SBP reduction reached -24.7 mmHg (95% CI: -26.2 to -23.3; p < 0.001), and DBP reduction reached -10.4 mmHg (95% CI: -11.6 to -9.2; p < 0.001). At 12 weeks, the mean SBP further declined to 127.4 ± 17.9 mmHg, corresponding to a total reduction of -29.3 mmHg (95% CI: -30.7 to -27.9; p < 0.001), while DBP dropped to 75.6 ± 10.2 mmHg, with a mean change of -12.9 mmHg (95% CI: -14.1 to -11.7; p < 0.001) (Table 5). Subgroup analyses showed comparable BP reductions across age groups and between treatment-naïve and previously treated patients, with no statistically significant differences observed (p > 0.05).

**Table 5:** Changes in systolic and diastolic blood pressure over time (n = 185) SBP: systolic blood pressure; DBP: diastolic blood pressure; mmHg: millimeters of mercury Values are presented as mean change with 95% confidence intervals (CI) compared with baseline. p-values are derived from paired t-tests; * indicates statistical significance at p < 0.05.

Time Point	SBP (mmHg)		DBP (mmHg)	
	Mean ± SD	Mean Change (95% CI; p-value)	Mean ± SD	Mean Change (95% CI; p-value)
Baseline	156.7 ± 19.7	–	88.5 ± 11.9	–
Week 2	135.8 ± 18.9	−20.9 (−22.1 to −19.7; <0.001)	80.4 ± 10.8	−8.1 (−9.3 to −7.0; <0.001)
Week 4	132.0 ± 18.2	−24.7 (−26.2 to −23.3; <0.001)	78.1 ± 10.4	−10.4 (−11.6 to −9.2; <0.001)
Week 12	127.4 ± 17.9	−29.3 (−30.7 to −27.9; <0.001)	75.6 ± 10.2	−12.9 (−14.1 to −11.7; <0.001)

Blood pressure changes in treatment-naive and previously treated patients

**Figure 2:**
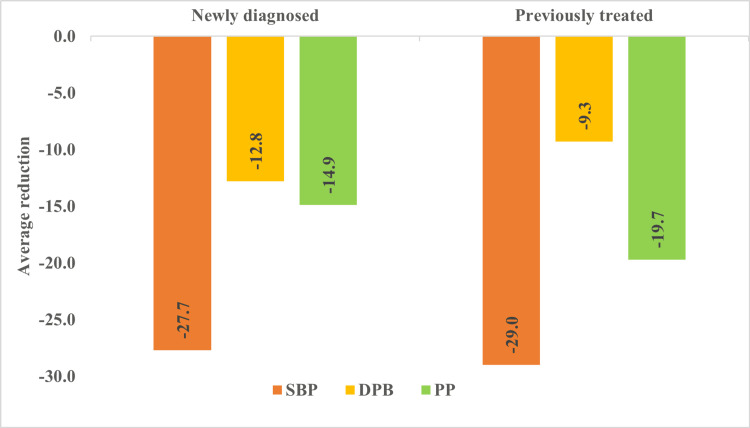
Blood pressure changes in treatment-naive and previously treated patients SBP: systolic blood pressure; DBP: diastolic blood pressure; PP: pulse pressure Average reductions in SBP, DBP, and PP after 12 weeks of indapamide/amlodipine therapy in newly diagnosed and previously treated patients with isolated systolic hypertension.

Both newly diagnosed and previously treated patients experienced significant BP reductions. SBP decreased slightly more in previously treated patients (-29.0 mmHg) than in newly diagnosed patients (-27.7 mmHg). However, DBP reduction was greater in newly diagnosed patients (-12.8 mmHg vs. -9.3 mmHg). PP reduction was more pronounced in previously treated patients (-19.7 mmHg vs. -14.9 mmHg) (Figure 2).

Achievement of the target SBP at each follow-up

**Figure 3:**
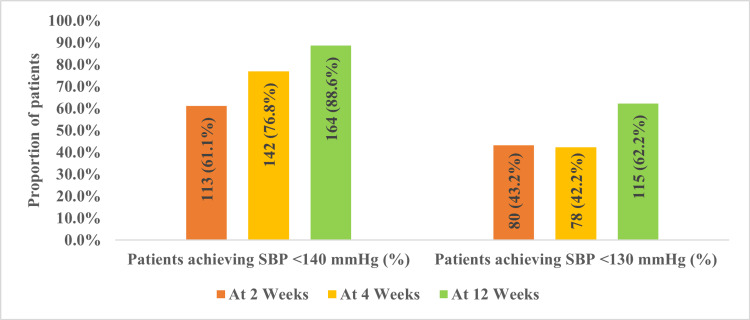
Proportion of patients achieving target SBP (<140 mmHg and <130 mmHg) over time SBP: systolic blood pressure; mmHg: millimeters of mercury Proportion of patients achieving SBP targets (<140 mmHg and <130 mmHg) at two, four, and 12 weeks of indapamide/amlodipine therapy. Values are presented as frequency (percentage); n=185.

The proportion of patients achieving SBP <140 mmHg increased progressively, with 113 (61.1%) at two weeks, 142 (76.8%) at four weeks, and 164 (88.6%) at 12 weeks. In contrast, 80 (43.2%) of patients reached SBP <130 mmHg at two weeks, 78 (42.2%) at four weeks, and 115 (62.2%) at 12 weeks (Figure 3).

Age-related BP response and treatment consistency at 12-week follow-up

**Figure 4:**
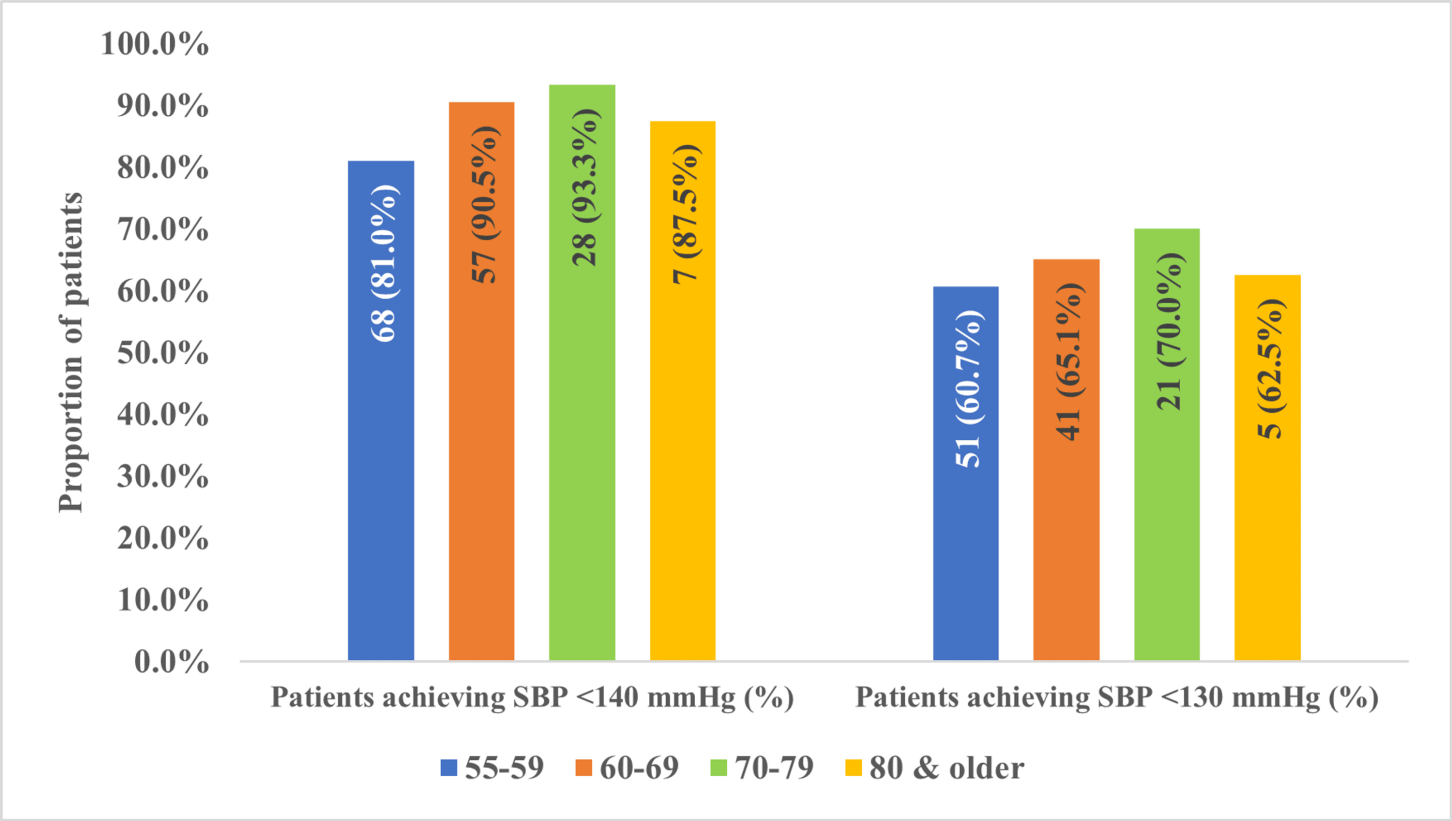
Achievement of target SBP at 12-week follow-up by age group SBP: systolic blood pressure; mmHg: millimeters of mercury Proportion of patients achieving SBP targets (<140 mmHg and <130 mmHg) at 12 weeks of indapamide/amlodipine therapy by age groups.

By the 12-week follow-up, more than 80% of patients in each age group achieved a SBP of less than 140 mmHg: 68 (81.0%) of patients in the 55-59 years group, 57 (90.5%) in the 60-69 years group, and seven (87.5%) in the 80 years and older group, with the highest proportion in the 70-79 years age group, responsible for 28 (93.3%) of patients achieving the target SBP. Furthermore, over 60% of patients in each age group reached an SBP of less than 130 mmHg: 51 (60.7%) of patients in the 55-59 years group, 41 (56.1%) in the 60-69 years group, and five (62.5%) in the 80 years and older group, with the highest success rate also in the 70-79 years group, accounting for 21 (70.0%) of patients. However, these variations were not statistically significant (p > 0.05) (Figure 4).

Metabolic and electrolyte effects

No significant changes were observed in serum sodium or creatinine levels over the 12-week follow-up period (p > 0.05). A small but statistically significant decrease in mean serum potassium was noted, from 4.05 ± 0.54 mmol/L at baseline to 3.87 ± 0.51 mmol/L at 12 weeks (p = 0.002). However, this change remained within the lower limit of the normal range and was not associated with any clinical symptoms. Four patients had potassium levels below 3.5 mmol/L, but none required clinical intervention (Table 6).

**Table 6:** Changes in serum electrolytes and creatinine levels over the study period SD: standard deviation. Values are expressed as mean ± SD Differences represent change from baseline to 12 weeks, with percentage change calculated relative to baseline. p-values are derived from paired t-tests. * indicates statistical significance at p < 0.05.

Variables	Baseline Mean ± SD	After 12 weeks Mean ± SD	Difference Mean ± SD	% Changes	p-value
Potassium	4.1 ± 0.5	3.9 ± 0.51	0.17 ± 0.58	4.2%	0.002*
Sodium	137.9 ± 3.4	137.5 ± 3.2	0.40 ± 4.27	0.29%	0.312
Serum creatinine	1.0 ± 0.3	1.0 ± 0.26	0.03 ± 0.22	3.0%	0.213

Tolerability and patient well-being

The indapamide/amlodipine combination therapy was generally well tolerated, with no serious adverse events (SAEs) reported. Mild adverse events occurred in a small proportion of patients, including leg edema in 3/185 (1.6%), hypokalemia in 4/185 (2.2%), and symptomatic hypotension in 1/185 (0.5%). VAS scores (mean ± SD) increased from 55.9 ± 17.0 at baseline, 68.2 ± 14.5 at two weeks, 72.7 ± 14.6 at four weeks, and 78.9 ± 14.1 at 12 weeks (p < 0.001).

## Discussion

This study suggests that the FDC of indapamide and amlodipine appears to be effective in managing isolated systolic hypertension (ISH) in patients aged 55 years and older. The observed reductions in SBP and DBP across all age groups indicate that this treatment approach may provide meaningful benefits in real-world practice. The safety and BP-lowering patterns observed in this study are generally consistent with findings from similar studies [18,19].

Over 12 weeks, the indapamide/amlodipine combination was associated with a rapid and sustained BP reduction, with most of the effect occurring within the first four weeks. By week 12, SBP and DBP declined by -29.3 mmHg and -12.9 mmHg, respectively, closely matching the reductions reported in the ARBALET study, which used the same regimen in older adults with ISH [17]. These findings likely reflect the complementary mechanisms of indapamide and amlodipine, which target arterial stiffness and vascular resistance commonly associated with ISH [5]. Additionally, subgroup analyses indicated consistent treatment effects across different age groups and prior treatment status, suggesting the combination’s broad applicability in older adults with ISH.

BP target achievement increased steadily over the 12 weeks, with nearly 90% of patients achieving SBP <140 mmHg by study end, consistent with prior trials of diuretic-calcium channel blocker (CCB) combinations, which have consistently demonstrated superior BP-lowering effects compared to monotherapy [20,21]. This early response is clinically relevant, as prompt BP control is associated with a lower risk of cardiovascular events, particularly in older adults with ISH [22,23].

Patients previously treated with antihypertensive agents exhibited slightly greater reductions in SBP compared with treatment-naïve patients (-29.0 mmHg vs. -27.7 mmHg). In contrast, newly diagnosed patients showed a greater reduction in DBP (-12.8 mmHg compared to -9.3 mmHg), indicating a stronger response to the initiation of therapy. Minor fluctuations in DBP between weeks two and four were noted in treatment-naïve patients, although these were not statistically significant. The more substantial pulse pressure reduction observed in previously treated patients may suggest a cumulative improvement in arterial compliance with long-term antihypertensive therapy, consistent with earlier findings of structural vascular remodeling over extended treatment periods in human studies [24,25]. Overall, the progressive reductions in BP, together with narrowing confidence intervals over the follow-up period, support the consistency of blood pressure control observed with this FDC in a real-world setting.

This study provides real-world evidence that the FDC of indapamide and amlodipine was effective in helping patients achieve target SBP <140 mmHg across all age groups, with over 80% achieving this goal at 12 weeks. The 70-79-year-old group had the highest achievement rate (93.3%), which is consistent with previous studies showing that diuretic-CCB combinations improve arterial compliance and reduce vascular resistance. The 55-59-year-old group had the lowest rate (80.95%), which may reflect higher baseline BP levels or differences in adherence. For the more stringent SBP target of <130 mmHg, the 70-79-year-old group again had the highest success rate (70.9%), while the ≥ 80-year-old group had the lowest (62.5%). This is likely due to greater arterial stiffness and reduced drug responsiveness in very elderly patients [26]. The consistently high SBP target attainment observed over 12 weeks suggests that the indapamide/amlodipine combination is a practical and well-tolerated treatment option for older adults with ISH, aligning with recommendations that favor diuretic-CCB combinations as first-line therapy [17,22]. However, given the observational design, these findings should be interpreted as associations rather than definitive causal effects.

Regarding electrolyte changes, the treatment did not induce significant changes in serum sodium or creatinine levels, reinforcing its renal safety profile. A mild but statistically significant decrease in potassium was observed (4.05 ± 0.54 to 3.87 ± 0.51 mmol/L; p = 0.002), consistent with prior research on indapamide’s mild diuretic effect [27]. However, this reduction remained within the lower normal range and had no clinical impact.

The indapamide/amlodipine combination was well tolerated, with adverse events occurring infrequently, with leg edema in 1.6%, mild electrolyte imbalance in 2.2%, and transient BP fluctuations in 0.5% of patients. No serious adverse events were reported. Moreover, self-reported well-being scores (VAS score) improved significantly over time, reflecting a meaningful enhancement in patients' perceived health status and quality of life. This improvement aligns with the observed reductions in blood pressure and is consistent with previous studies [27,28].

Clinical Implications

These findings highlight the clinical value of a diuretic-CCB combination as a first-line or adjunct therapy for ISH in older adults. The high BP control rates, minimal side effects, and improved patient satisfaction support its real-world effectiveness in hypertension management. However, treating elderly patients with long-standing severe ISH requires caution, as rapid BP reduction may pose risks [29]. Since no single regimen is universally preferred, treatment decisions should be based on target organ damage, vascular risk, drug tolerability, clinical response, and comorbid medication interactions [30].

Study limitations and future directions

This study has several limitations that should be taken into account when interpreting the findings. First, the observational and open-label design without a control group limits causal inference and introduces potential biases, such as regression to the mean and the Hawthorne effect. A control group was not included due to both practical and ethical considerations, as withholding treatment from older adults with uncontrolled ISH would have posed unacceptable risks in routine clinical care. Instead, the study focused on real-world outcomes of active therapy to better reflect clinical practice. Second, the relatively short 12-week follow-up prevents assessment of the long-term durability of blood pressure control and cardiovascular outcomes. Third, although standardized training and SOPs were used, slight variations in BP measurement techniques across centers could have caused inter-site variability. Finally, relying on self-reported VAS scores for well-being may introduce reporting bias. These findings should be interpreted as associations rather than causal effects, as the uncontrolled, observational design cannot fully exclude the influence of factors such as regression to the mean, placebo response, or seasonal variations. Future research should include randomized controlled designs with longer follow-up durations, larger sample sizes to enhance safety assessments, and the incorporation of home or ambulatory blood pressure monitoring to provide a more comprehensive understanding of treatment effects and variability in real-world settings.

## Conclusions

The FDC of indapamide and amlodipine was associated with substantial reductions in SBP and DBP and was generally well tolerated in this cohort of older adults with isolated systolic hypertension. While these findings suggest potential clinical benefits, they should be interpreted cautiously given the uncontrolled, observational design. Further studies are needed to validate these results and explore long-term cardiovascular outcomes.
